# Rapid identification of pathogens, antibiotic resistance genes and plasmids in blood cultures by nanopore sequencing

**DOI:** 10.1038/s41598-020-64616-x

**Published:** 2020-05-06

**Authors:** Arne M. Taxt, Ekaterina Avershina, Stephan A. Frye, Umaer Naseer, Rafi Ahmad

**Affiliations:** 1 Department of Microbiology, Division of Laboratory Medicine, Oslo University Hospital, PB 4956, Nydalen 0424 Oslo, Norway; 2 Department of Biotechnology, Inland Norway University of Applied Sciences, Holsetgata 22, 2317 Hamar, Norway; 3 Department of Zoonotic, Food- and Waterborne Infections, 0213 Oslo, Norwegian Institute of Public Health, Oslo, Norway; 4 Institute of Clinical Medicine, Faculty of Health Sciences, UiT - The Arctic University of Norway, Hansine Hansens veg 18, 9019 Tromsø, Norway

**Keywords:** Genome informatics, Computational biology and bioinformatics, Genomic analysis, Next-generation sequencing, Microbiology, Clinical microbiology, Infectious-disease diagnostics

## Abstract

Bloodstream infections (BSI) and sepsis are major causes of morbidity and mortality worldwide. Blood culture-based diagnostics usually requires 1–2 days for identification of bacterial agent and an additional 2–3 days for phenotypic determination of antibiotic susceptibility pattern. With the escalating burden of antimicrobial resistance (AMR) rapid diagnostics becomes increasingly important to secure adequate antibiotic therapy. Real-time whole genome sequencing represents a genotypic diagnostic approach with the ability to rapidly identify pathogens and AMR-encoding genes. Here we have used nanopore sequencing of bacterial DNA extracted from positive blood cultures for identification of pathogens, detection of plasmids and AMR-encoding genes. To our knowledge, this is the first study to gather the above-mentioned information from nanopore sequencing and conduct a comprehensive analysis for diagnostic purposes in real-time. Identification of pathogens was possible after 10 minutes of sequencing and all predefined AMR-encoding genes and plasmids from monoculture experiments were detected within one hour using raw nanopore sequencing data. Furthermore, we demonstrate the correct identification of plasmids and *bla*_CTX-M_ subtypes using *de novo* assembled nanopore contigs. Results from this study hold great promise for future applications in clinical microbiology and for health care surveillance purposes.

## Introduction

Bloodstream infections (BSIs) and sepsis are major causes of morbidity and mortality worldwide. Epidemiological data are scarce, but a recent estimate indicated that 31.5 million cases of sepsis and 5.3 million sepsis attributable deaths occur annually^1^. This estimate is only based on data collected from high-income countries, and it therefore likely underestimates the true burden of disease worldwide, especially in low-and-middle-income countries^2^. Most studies on sepsis and BSIs report an increasing incidence over the last two decades^3^, particularly among the immunocompromised, multimorbid, and elderly patients, or due to failure of empiric antibiotic regimens as result of antimicrobial resistance (AMR)^4^.

With multi drug resistant pathogens spreading at an alarming rate, widely adopted empirical antibiotic treatment regimens for sepsis based on penicillin (or aminopenicillin) in combination with gentamicin^5^ are being challenged. In particular, the escalating burden of infections due to extended-spectrum β-lactamase (ESBL) producing Gram negative bacteria represents a major health concern. These bacteria, mainly *Escherichia coli* and *Klebsiella pneumoniae*, are not only resistant to all penicillins and third generation cephalosporins, but also frequently express co-resistance to gentamicin. Consequently, treatment failure may occur, and clinicians increasingly prescribe last-resort antibiotics such as carbapenems as initial antibiotic treatment of sepsis. This in turn contributes to development and spread of AMR and to a further increase in the burden of infections caused by resistant bacteria.

Current state-of-the art in diagnostics of BSIs is blood culture, which often takes 1–3 days to come out positive and provide information on etiological agent. Time to positivity is influenced by a number of clinical and microbiological factors such as source of bacteraemia, level of bacteraemia, presence or absence of pre-administered antibiotics and the bacterial species^6^. Use of matrix-assisted laser desorption/ionization time-of-flight (MALDI-TOF) mass spectrometry is becoming widespread in clinical microbiology laboratories to identify bacteria by analysis of pelleted blood-cultures when they are flagged as positive by the blood culture incubation system^7^. Phenotypic antimicrobial susceptibility testing (AST) however, requires subculture on solid media overnight for colonies to form, and an additional 18+/−2 hours of subculture incubation with antibiotic discs to obtain a result which can be interpreted according to official breakpoint guidelines and converted to sensitive (S), intermediate (I) or resistant (R)^8^. Until then time-point choice of antibiotic treatment is based on clinical assessment, empirical guidelines and local epidemiology on AMR. However, several studies have observed that inappropriate antibiotic treatment is often initiated to patients with BSIs and this is associated with increased mortality^9,10^.

Diagnosis of BSI and prescription of appropriate antimicrobial therapy is crucial for the reduction of morbidity and mortality caused by BSI and WHO and Centers for Disease Control aim for a two hour turnaround time^11^. For these reasons there is a growing interest and an urgent need for the development of molecular techniques for rapid identification of pathogens and AMR from blood and blood cultures. The topic has been extensively reviewed elsewhere^12–14^, and the currently available methods can be categorized in three groups; *in situ* hybridization-based methods, DNA-microarray-based methods, nucleic acid amplification-based methods (such as PCR and loop-mediated isothermal amplification (LAMP)), and combinations of these. Common to all these techniques is that detection is limited to a predefined set of genetic targets, either specific for a particular pathogen or an AMR-encoding gene. Whole genome sequencing (WGS) on the other hand, provides comprehensive genomic information and can potentially detect all AMR-encoding genes present in the bacterial genome. Additionally, WGS-data provide vast opportunities for bacterial sequence typing, phylogenetics and virulence analysis. The introduction of the real-time sequencing platform from Oxford nanopore technology (ONT) has triggered studies to explore its application in blood culture diagnostics, either based on 16 s amplicon sequencing^15^, or by a whole-genome-sequencing approach^16^. This represents an unbiased approach to diagnostics with the potential to identify any pathogen and AMR-encoding gene.

Here we present results from rapid blood culture diagnostics based on extraction of bacterial DNA from positive blood cultures followed by nanopore sequencing and real-time data analysis for identification of pathogens, detection of plasmids and AMR-encoding genes. The results have also been verified through WGS using short-read Illumina sequencing and hybrid assembly using nanopore and Illumina sequences. This proof-of-concept study represents a molecular-genetic approach to diagnosis of BSIs which can provide clinicians with detailed information on etiologic agent and AMR within few hours of a blood culture becoming positive.

## Results

### Blood culture samples and nanopore sequencing

Seven blood cultures spiked with *bla*_CTX-M_ positive *E. coli* and *K. pneumoniae*, *mecA* positive *Staphylococcus aureus*, or a combination of these were analysed. In addition, one blood culture was spiked with *E. coli* reference strain CCUG17620 (non-ESBL) as control. All cultures were incubated in a standard BD BACTEC FX blood culturing instrument with continuous monitoring until flagged positive. Positive monocultures had bacterial concentrations ranging from 2.6 × 10^7^ to 1.6 × 10^9^ CFU/ml. When *E. coli* and *S. aureus* were co-cultured, we observed a 4-log difference in bacterial concentration in favour of *E. coli*, while for the *E. coli* and *K. pneumoniae* co-culture the difference was less than one-log fold in favour of *K. pneumoniae* (Table 1).

**Table 1:** Overview of blood culture experiments.

Nr	Bacteria	AMR gene	CFU/ml	DNA [ng/µl]
1	*E. coli (CCUG17620)*	*−*	1,3 × 10^9^	60
2	*E. coli (A2-39)*	*CTX-M-2*	1,6 × 10^9^	57
3	*E. coli (NCTC13441)*	*CTX-M-15*	1,7 × 10^9^	49
4	*K. pneumoniae(A2-23)*	*CTX-M-1*	9,3 × 10^8^	54
5	*K. pneumoniae(A2-37)*	*CTX-M-14*	1,0 × 10^9^	56
6	*S. aureus(CCUG35600)*	*mecA*	2,6 × 10^7^	31
7	*E. coli (A2-239)*	*CTX-M-2*	5,5 × 10^7^	45
	+		+	
	*K. pneumoniae (A2-37)*	*CTX-M-14*	4,8 × 10^8^	
8	*E. coli (NCTC13441)*	*CTX-M-15*	1,2 × 10^9^	54
	+		+	
	*S. aureus (CCUG35600)*	*mecA*	3,6 × 10^5^	

For extraction of bacterial DNA, the commercially available kits MolYsis Plus and BiOstic Bacteremia DNA were both used initially. Subsequent experiments were conducted using the BiOstic Bacteremia DNA kit due to its shorter protocol and higher DNA yield (Supplementary Fig. 1). Purified DNA was subject to nanopore sequencing on the MinION sequencing platform from ONT. The average amount of data generated was 334,113 ± 379,926 (mean ± standard deviation) sequencing reads per blood culture with an average read length of 3,529 ± 4,140 bp (Supplementary Fig. 2). Only 15.4% ± 14.4% of the reads were shorter than 300 bp, which is the maximum single-end read length generated by the latest version of the Illumina MiSeq system.

### Bacterial species identification using raw nanopore sequencing reads

Using default settings, the Metrichor analysis platform from ONT performed real-time base calling generating 4,000 sequences per output file. For taxonomy assignment, both Centrifuge^17^ classification (used by the ONT analysis tool What´s In My Pot (WIMP)^18^) and BLAST search against the NCBI Prokaryotic RefSeq (RefProk) database took less than one minute per sequencing file using four cores, which is the standard configuration on current laptops. We hereby present detailed results for two monoculture experiments, *E. coli* A2-39 and *K. pneumoniae* A2-37, and for one blood culture spiked with both of these isolates.

**Figure 1:**
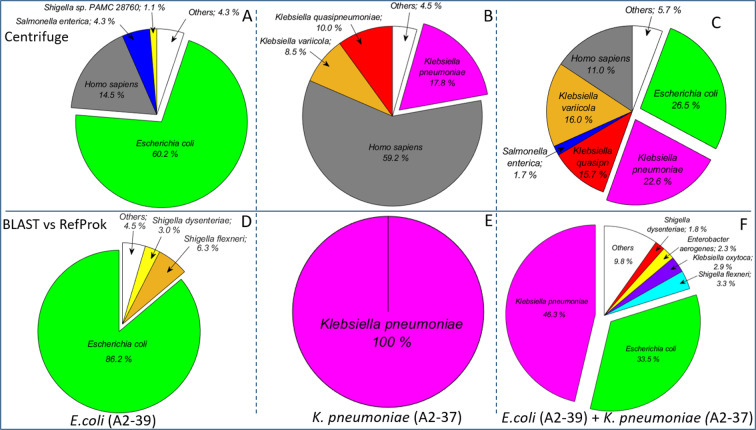
Relative distribution of reads in sequence data generated by nanopore sequencing of DNA purified from the three selected blood cultures. The blood cultures were spiked with *E. coli* A2-39 (**A** and **D**), *K. pneumoniae* A2-37 (**B** and **E**) and *E. coli* A2-39 + *K. pneumoniae* A2-37 (**C** and **F**). Upper panel (**A**–**C**) show results obtained using Centrifuge and lower panel (**D**–**F**) show results based on BLAST search against the RefProk database that contains prokaryotic sequence data only. The “Others” group represents taxa with relative read counts below 1%. All results are based only on the first output file for each experiment from the MinION sequencing platform, containing 4000 reads (available after approximately 10 minutes of sequencing).

**Figure 2:**
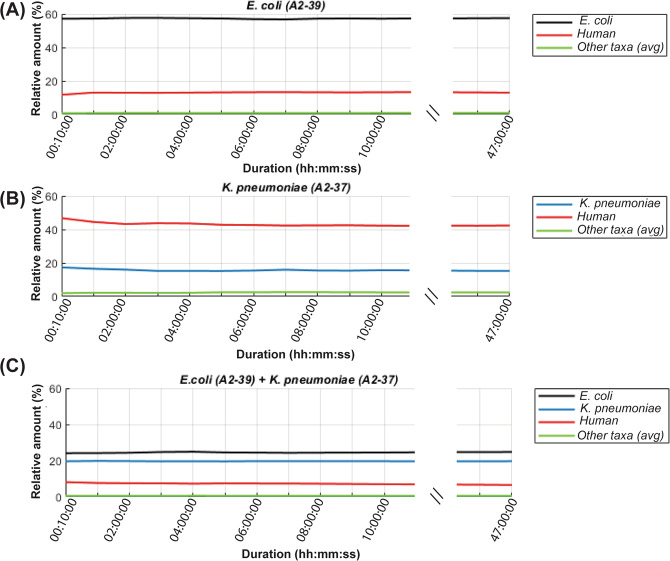
Centrifuge-based relative taxonomic assignments of sequence data from DNA purified from the three selected blood cultures. Samples were spiked with - (**A**) *E. coli* A2-39, (**B**)* K. pneumoniae* A2-37 and (**C**) *E. coli* A2-39 + *K. pneumoniae* A2-37. The “Others” group shows the average relative amount of all incorrectly assigned (non-target) taxa. Blood cultures spiked with *E. coli* (A2-39) and with a combination of *E. coli* (A2-39) and *K. pneumoniae* (A2-37) were sequenced for 68 and 64 hours respectively.

Based only on the first sequence file 80–100% of bacterial reads were classified correctly at the species level by BLAST search against the RefProk database (Fig. 1D–F) and most of the ‘incorrect’ assignments, i.e. reads classified as non-target bacteria, comprised less than 1% per species (Fig. 2A–C). Also, relative amounts of the detected species remained constant throughout the sequencing run (Fig. 2A–C).

Intriguingly, 4.3% of sequencing reads from *E. coli* A2-39 monoculture and 1.7% of reads from the *E. coli* A2-39 + *K. pneumoniae* A2-37 mixed culture were classified as belonging to *Salmonella enterica* by Centrifuge, but not by BLAST (Fig. 1A,C,D and F). Following a detailed analysis, the majority of these misclassified reads were identified as *E. coli* specific sequences using BLAST against the RefProk and RefSeq databases (Supplementary Fig. 3). The average nucleotide identity (ANI) values of these reads were 79.6% for *Salmonella enterica* (AE014613.1) and 83.6% for *E*. *coli* (NC_000913.3). Around 9% of *E*. *coli* A2-39 culture reads were classified as *Shigella* by BLAST search against RefProk database, with ANI values of 87.9% for *Shigella flexneri* (AE014073.1) and 88.8% for *E. coli* (NC_000913.3).

In both analyses of the *K. pneumoniae* A2-37 isolate, in monoculture and in the *E. coli* A2-39 + *K. pneumoniae* A2-37 mixed culture, *K. pneumoniae* A2-37 reads were evenly classified as *K. pneumoniae*, *K. quasipneumoniae* or *K. variicola* by Centrifuge (Fig. 1B,C). However, BLAST search against RefProk classified all of these reads as *K. pneumoniae* (Fig. 1E,F). ANI values for these reads were on average 85.2% for reads assigned to *K. pneumoniae* (NC_016845.1), 84.2% for reads assigned to *K. variicola* (NZ_CP010523.2) and 87.0% for reads assigned to *K. quasipneumoniae* (NZ_CP014696.2).

The other four monoculture experiments of *E. coli, K. pneumoniae* and *S. aureus* also showed very promising results, with 94-100% of reads assigned correctly to target species using the first 4000 reads (Supplementary Fig. 4). In the mixed culture experiment with *E. coli* and *S. aureus* however, 94.7% of sequences were assigned to *E. coli* and only 0.05% of reads were assigned to *S. aureus* based on the first sequence file. Throughout the entire sequencing run the relative number of reads classified as *S. aureus* remained below 1% and were thereby indistinguishable from other low-level misassignments. A probable explanation could be the 4-log difference in bacterial concentration in favour of *E. coli* in the experiment (Table 1).

### Detection of AMR-encoding genes and plasmids using raw nanopore sequencing reads

**Figure 3:**
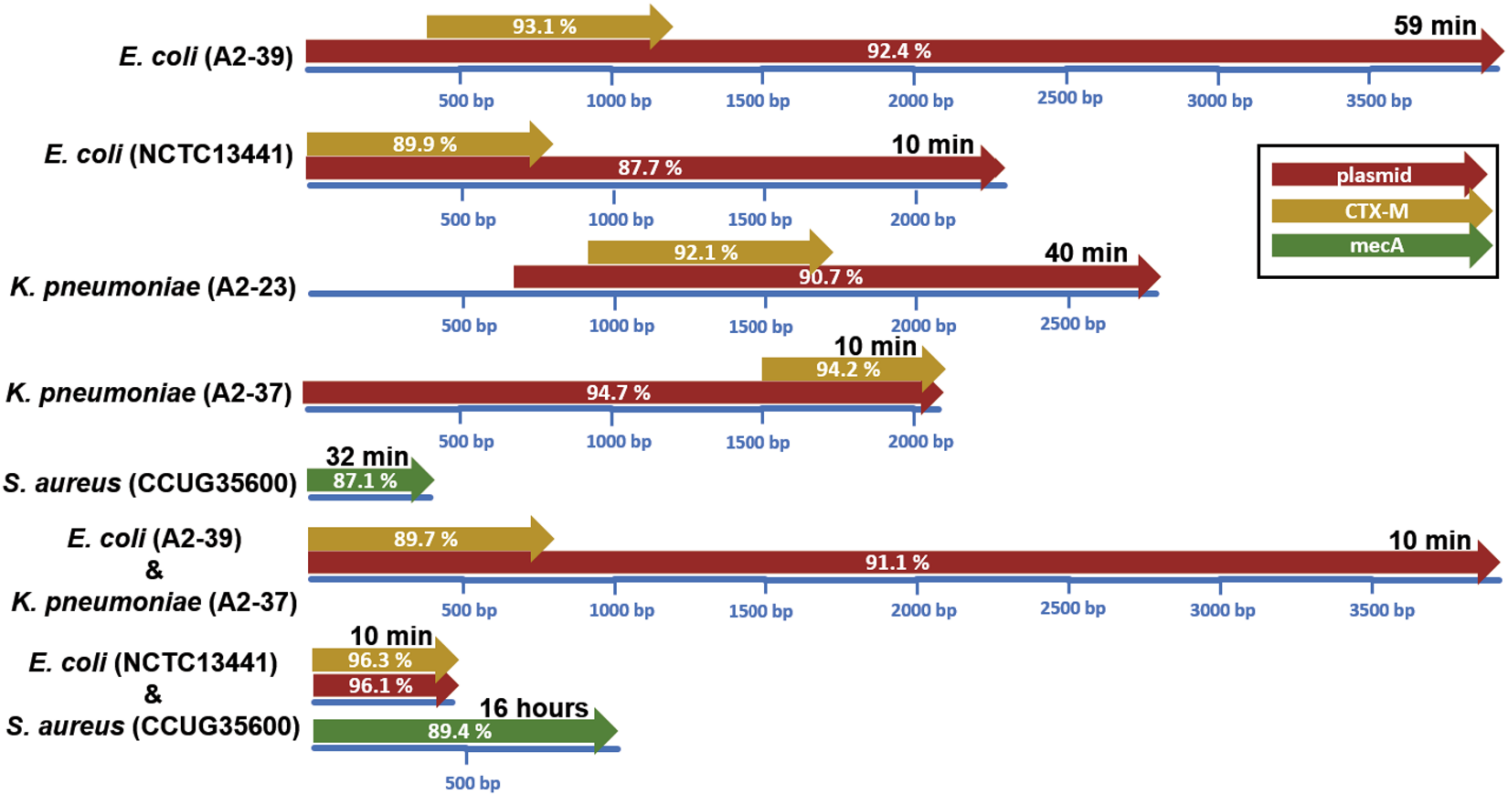
Overview of the earliest generated reads from each sequencing experiment where *bla*_CTX-M_ (yellow) or *mecA* genes (green) and plasmid-derived sequences (red) were identified based on BLAST search. Blue lines represent length of the nanopore reads, length of hits is illustrated by arrow-length and similarity to the database entry is given on the arrow. The time of read generation is noted on top of each read.

BLAST search against the CARD and ResFinder databases showed similar AMR gene assignments and therefore these results are presented together. In case of the reference strain (*E.coli* CCUG17620) spiked blood culture none of the reads were identified as *bla*_CTX-M_ positive. For all monocultures of *bla*_CTX-M_ or *mecA*-positive bacteria, target AMR genes were detected within the first hour of sequencing (Fig. 3). However, the unassembled sequencing data did not allow correct identification of *bla*_CTX-M_ gene variant (Supplementary Fig. 5). Sequencing reads containing *bla*_CTX-M_ genes were recognized as plasmid-borne when BLAST search of these reads against the plasmid database from Brooks *et al*.^19^ yielded positive hits (Fig. 3). The *mecA* positive reads from the *S. aureus* spiked blood culture were not detected in the plasmid database and therefore were labelled as chromosome borne. The first *bla*_CTX-M_ containing read from the *E. coli* A2-39 + *K. pneumoniae* A2-37 spiked blood culture was detected 10 minutes after the start of sequencing. Ten minutes were also enough to capture the first *bla*_CTX-M_ containing read from the *E. coli* NCTC 13441 + *S. aureus* CCUG35600 spiked blood culture, but the first indication of the *mecA* gene came only after 16 hours.

### *De novo* assembled nanopore contigs enable identification of plasmids and *bla*_CTX-M_ gene-variants

Using *de novo* assembled contigs from *E. coli* A2-39 and *K. pneumoniae* A2-37 monoculture experiments we searched for plasmids with the NCBI PlasmidFinder tool. *E.coli* A2-39 harboured IncHI2, IncI1 and p0111 plasmids, whereas *K. pneumoniae* A2-37 harboured IncFII and IncFI plasmids (Table 2). Assembled contigs from sequencing of the blood culture spiked with both isolates also suggested presence of these plasmids. By performing additional BLAST searches with the plasmid-labelled contigs against the AMR databases the *bla*_CTX-M_ gene-variants could be identified (Table 2).

**Table 2:** BLAST search results of contigs harbouring plasmids against the AMR databases. HSP – high scoring segment pair.

Bacterial culture	PlasmidFinder			Resistance genes database			
	Plasmid type	Query/HSP length, bp	Identity, %	CTX-M variant	Hit length, bp	Identity, %	E-value
***E. coli (*A2-39)**	*IncHI2*	327/327	100	*CTX-M-2*	878	98.7	0
	*IncI*1	142/142	99.3				
	*p0111*	885/885	98.9				
***K. pneumoniae (*A2-37)**	*IncFII*	231/230	98.3	*CTX-M-14*	877	99.5	0
	*IncFIA*	388/388	97.2				
	*IncFIB*	512/560	98.2				
***E. coli (*A2-39) +*K. pneumoniae*** **(A2-37)**	*IncHI2*	630/630	99.2	*CTX-M-2*	877	99.3	0
	*p0111*	885/885	98.8				
	*IncFIA*	388/388	96.9	*CTX-M-14*	876	100	0
	*IncFIB*	560/560	98.7				
	*IncFII*	261/261	100				

To assess whether the first raw sequencing reads that were recognized as containing *bla*_CTX-M_ were plasmid-borne, they were mapped to the *de novo* assembled contigs. The *E. coli* A2-39 *bla*_CTX-M_-containing read detected after 59 minutes of sequencing mapped to the IncHI2-tagged contig with 92.1% identity (length = 3,961 bp; query coverage = 100%; e-value = 0), whereas the *K. pneumoniae* A2-37 *bla*_CTX-M_ containing read detected after 10 minutes of sequencing mapped to the IncFII-tagged contig with 94% identity (length = 2,055 bp; query coverage = 100%; e-value = 0). The first *bla*_CTX-M_ containing read from the *E. coli* A2-39 + *K. pneumoniae* A2-37 spiked blood culture that was detected after ten minutes of sequencing, mapped only to the IncFII-tagged *K. pneumoniae* contig with 91.2% identity (length = 3 840 bp; query coverage = 100%; e-value = 0). The first read that mapped to the IncHI2-tagged *E. coli* contig, was detected in the second output file generated after 36 minutes of sequencing (length = 4 178 bp; query coverage = 99.9%; identity = 91.2%; e-value = 0).

### Identification of bacterial species, AMR-encoding genes and plasmids using raw Illumina sequencing reads

To verify taxonomic classification, AMR-gene detection and plasmid identification, the *E. coli* A2-39 and *K. pneumoniae* A2-37 isolates were sequenced on an Illumina MiSeq platform. Taxonomic assignment of the unassembled Illumina reads by Centrifuge was similar to the results obtained with nanopore data; where 4.7% of the *E. coli* A2-37 reads were assigned as *S. enterica*, and *K. pneumoniae* reads were assigned as a combination of *K. pneumoniae*, *K. quasipneumoniae* and *K. variicola* group (Supplementary Fig. 6). For detection of AMR-encoding genes, CARD and ResFinder databases were searched using SPAdes assembled contigs (see Supplementary Table 1 for details). This suggested that *E. coli* A2-39 harboured an IncHI2 plasmid encoding the *bla*_CTX-M-2_ gene (hit length = 876 bp; identity = 100%; e-value = 0) and that *K. pneumoniae* A2-37 contained an IncFII plasmid with the *bla*_CTX-M-14_ gene (hit length = 876 bp; identity = 100%; e-value = 0).

### Hybrid *de novo* assembled data corroborate nanopore results

**Figure 4:**
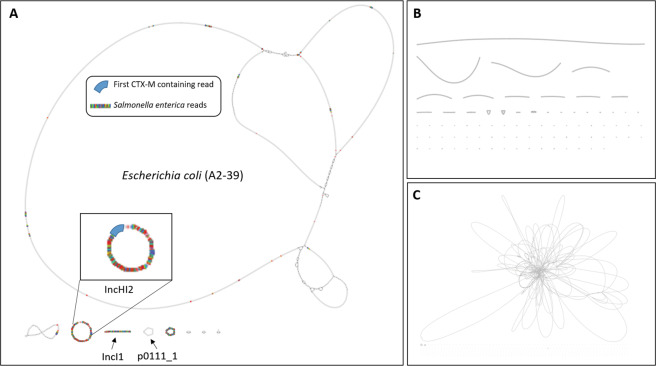
Assembly graphs for *E.* *coli* A2-39. (**A**) Hybrid assembly of nanopore and Illumina data; reads tagged as *S. enterica* by Centrifuge and *bla*_CTX-M_ are highlighted (*S. enterica* in multicolour, *bla*_CTX-M_ in blue). The *bla*_CTX-M_ gene was located on a plasmid-derived read which mapped to the IncHI2 plasmid. (**B**) Unicycler assembled nanopore generated data. (**C**) SPAdes assembled Illumina generated data.

Hybrid *de novo* assembly using Illumina reads and nanopore reads from blood cultures spiked with *E. coli* A2-39 or *K. pneumoniae* A2-37 was performed using Unicycler (Fig. 4 and Supplementary Table 1). Generally, hybrid assembled data corroborated results obtained using nanopore assembled data only, albeit with higher precision. The *bla*_CTX-M-2_ gene variant was detected in *E. coli* A2-39 (identity = 100%; hit coverage = 100%; e-value = 0) and *K. pneumoniae* A2-37 was found to carry *bla*_CTX-M-14_ (identity = 100%; hit coverage = 100%; e-value = 0). Three contigs were tagged as plasmid-borne by PlasmidFinder (Supplementary Table 2). Additional search with these contigs against the PLSDB database revealed that one circular contig of the hybrid *E. coli* A2-39 assembly (length = 231,378 bp) had 99.8% identity to the IncHI2A *E. coli* RCS77_p plasmid (LT985297.1). The other circular contig (length = 95,977) had 98.0% identity to the p0111_1 *Enterobacteriaceae* plasmid (NZ_CP033848.1) and a non-circular contig had 99.6% identity to the IncI1 *E. coli* pS51_1 plasmid (NZ_CP015996.1).

One circular contig of the *K. pneumoniae* A2-37 hybrid assembly (length = 84,474 bp; depth = 2.41×) labelled as IncFII by PlasmidFinder had 99.8% identity to the IncFII *E. coli* pFAM22321 plasmid (KU288634.1). Two non-circular contigs of the *K. pneumoniae* A2-37 hybrid assembly (length = 128,678 bp and length = 121,056 bp) were recognized by PlasmidFinder as plasmids IncFIA(HI1) and IncFIB(K) (Supplementary Table 2). Additional search of these contigs through the PLSDB database showed that they had 93.8% identity to *K. pneumoniae* pKp_Goe_414-3 plasmid (NZ_CP018340.1) and 96.6% identity to *K. pneumoniae* strain AR_0049 unitig_2 plasmid (NZ_CP018818.1) respectively.

The first *bla*_CTX-M_ containing read from the *E. coli* A2-39 monoculture detected after one hour of sequencing mapped to the IncHI2 plasmid contig with 92.4% identity (length = 4,100 bp; query coverage = 100%; e-value = 0). The first *bla*_CTX-M_ containing read from the *K. pneumoniae* A2-37 monoculture detected after 10 minutes of sequencing mapped to the IncFII contig with 94.8% identity (length = 2,109 bp; query coverage = 100%; e-value = 0). Similar to the assembled nanopore data, the first *bla*_CTX-M_ tagged read from the *E. coli* A2-39 + *K. pneumoniae* A2-37 spiked blood culture mapped to the *K. pneumoniae* A2-37 hybrid assembly with 91.2% identity (length = 4,005 bp; query coverage = 100%; e-value = 0), but not to the *E. coli* A2-39 hybrid assembly. The first *bla*_CTX-M_ tagged read that mapped to the *E. coli* A2-39 hybrid assembly (length = 4,349 bp; query coverage = 99.9%; identity = 91.0%; e-value = 0), was found in the second output file generated 36 minutes after the sequencing start.

### Plasmid sequence analysis suggests horizontal gene transfer between *Salmonella species* and *E. coli*

Interestingly, 52.2% of the reads that were identified as *S. enterica* by Centrifuge in the *E. coli* A2-39 experiment mapped to plasmid-tagged contigs. Two thirds of these reads (1117 out of 1917) mapped to the *bla*_CTX-M-2_ harbouring IncHI2A plasmid contig (Fig. 4A–C). Moreover, this contig showed 99.7% identity to IncHI2 plasmids isolated from *S. enterica* subsp. *enterica* serovar Enteritidis strains (KM396300.1; KM396299.1 and KM396298.1), suggesting horizontal gene transfer between *Salmonella species* and *E. coli*. Of the *Shigella*-tagged reads from the same experiment the majority (81.6%) were evenly distributed across *E. coli* A2-39 chromosomal contigs and 18.4% mapped to plasmid contigs, mainly to the IncHI2A plasmid contig (929 out of 950 reads) (Supplementary Fig. 7).

### Eight hours of nanopore sequencing is sufficient for 95% genome coverage

**Figure 5:**
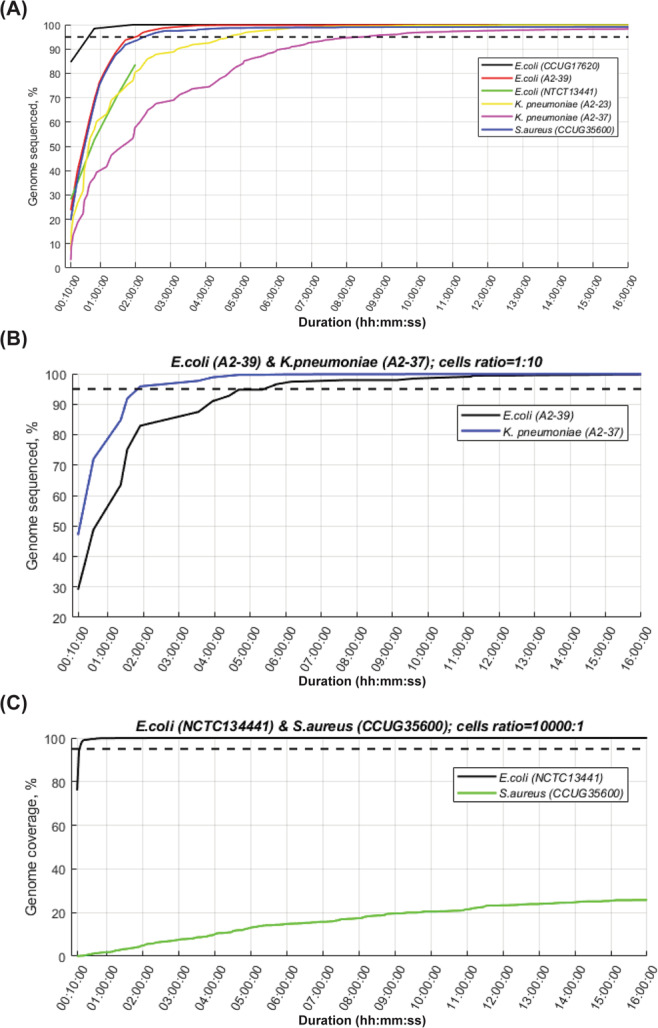
Genome coverage of target species over time. (**A**) Blood cultures spiked with monocultures. Reference strain *E. coli* (NCTC13441) experiment was stopped after 2 hours; at that point it reached 84% of the genome coverage. (**B**) Blood culture spiked with a combination of *E. coli* (A2-39) and *K. pneumoniae* (A2-23) (**C**) blood culture spiked with a combination of *E. coli* (NCTC13441) and *S. aureus* (CCUG35600). *S. aureus* reached 35% coverage by the end of the sequencing run of 33 hours. Dashed horizontal line denotes 95% genome coverage.

For the majority of monocultures, there was a steep increase to 95% genome coverage within the first two-four hours of sequencing (Fig. 5). Then accumulation of new information slowed down and genome coverage reached 99.0–99.8% after six hours of sequencing. In case of the *K. pneumoniae* A2-37 monoculture, which had more than 50% of human reads, it took eight hours to reach 95% genome coverage, 20 hours to reach 98.5% and 44.5 hours to reach 99.0% genome coverage (Fig. 5A). For the *E. coli* (A2-39) and *K. pneumoniae* (A2-37) spiked blood culture, it took only 2 hours to cover 95% of the *K. pneumoniae* (A2-37) genome as opposed to 5 hours to reach 95% coverage of *E. coli* (A2-39) genome (Fig. 5B). For the *E. coli* (NCTC13441) and *S. aureus* (CCUG35600) spiked blood culture, where the difference in bacterial concentration comprised 4-log, the *E. coli* (NCTC13441) genome was fully covered within the first hour of sequencing, whereas at 16 hours, when the *mecA* gene was detected, sequencing reads comprised information only on 25% of the *S. aureus* (CCUG35600) genome (Fig. 5C).

## Discussion

In this proof-of-concept study we present a sequencing-based approach to blood stream infection diagnostics that can identify pathogens and detect AMR-encoding genes within four hours from the time point when a blood culture is flagged as positive. By rapidly providing clinicians with bacterial pathogen identification and alerting them to the presence of clinically relevant AMR-encoding genes, this can contribute to early optimization of antibiotic therapy. Firstly, such an approach can save lives by triggering a change in antibiotic treatment in cases where inadequate therapy is given. Secondly, when established and validated further, this approach may contribute to early de-escalation of broad-spectrum therapy and antibiotic stewardship when no clinically relevant AMR-encoding genes are detected. Genotypic sequencing-based blood-culture diagnostics therefore represents a promising supplement to conventional phenotypic antimicrobial susceptibility testing.

In this study, we have shown that by use of the first 4000 raw sequencing reads, which become available after approximately 10 minutes of real-time sequencing, we can identify pathogens with a high degree of certainty. However, results obtained with Centrifuge (which is also utilized by the ONT analysis software WIMP) and search by BLAST differed substantially. Centrifuge is a very rapid tool for the classification of DNA sequences from microbial samples, which uses an indexing scheme optimized for the metagenomic classification. Here we have used the bacteria, archaea, virus and human indexing scheme for Centrifuge, which is derived from the NCBI Reference sequences (RefSeq) database. For the BLAST searches we used the NCBI Reference Prokaryotic (RefProk) database, which only contains prokaryotic genomic data. For this reason, human sequences are only recognized with the former approach. Using Centrifuge 4.3% of *E. coli* A2-39 reads were classified as *S. enterica*, and around half of these reads mapped to different plasmids (one third mapped to the IncHI2 plasmid), while the rest mapped to the *E. coli* chromosome. Since these sequences were correctly assigned to *E. coli* by the RefProk database search, possibly some of this *E. coli* genomic information is lacking in the indexed Centrifuge database, or misclassification is caused by the Centrifuge algorithm. The misclassification of the plasmid-sequences by Centrifuge highlights how mobile genetic elements may pose a challenge to sequence-based identification of bacteria. Bacterial identification should primarily be based on chromosomal gene content. Centrifuge also assigned reads from the *K. pneumoniae* A2-37 experiment to three different subspecies of *Klebsiella*; *K. pneumoniae*, *K. quasipneumoniae* and *K. variicola*, whereas BLAST search against RefProk classified 100% of reads as originating from *K. pneumoniae*. Also, these reads were classified correctly as *K. pneumoniae* when using BLAST search against the RefSeq database, indicating that the misclassification was potentially due to the Centrifuge search or indexing algorithm and not the RefSeq database.

For our monoculture experiments, classification by BLAST against RefProk assigned 100% of reads correctly for *K. pneumoniae* and *S. aureus*, whereas for the four *E. coli* experiments a slight proportion (from 3.1% to 9.3%) where assigned to *Shigella spp*. The distinction between *Shigella spp*. and *E. coli* is a well-known challenge to clinical microbiology since they share many biochemical, phenotypic and genetic properties. *Shigella* is widely believed to have evolved from *E. coli* and the genus *Shigella* comprises several clusters interspersed in the *E. coli* phylogeny^20^. Also, mapping of the *Shigella* tagged reads to *E. coli* A2-39 showed the reads to be distributed across the chromosome (Supplementary Fig. 6). Moreover, only 9% of these *Shigella*-tagged reads had more than 80% similarity to the essential *E. coli* genes downloaded from the database of essential genes^21^. The *Shigella* virulence plasmid, which is the key molecular signature of *Shigella spp*., was not found. For the differentiation between *E. coli* and *Shigella*, plasmid-encoded genetic information may be essential.

For this study we selected *bla*_CTX-M_ carrying strains of *E. coli* and *Klebsiella*, and a *mecA* positive strain of *S. aureus* for generation of mock blood-culture samples. These pathogens were selected because they are among the most frequently isolated bacteria from BSIs. Furthermore, the ESBL phenotype of *Enterobacteriaceae* and the MRSA phenotype of *S. aureus* are spreading globally at an alarming rate and are on the World Health Organization’s list of priority pathogens for which new antibiotics is urgently needed. In our monoculture experiments these target AMR-encoding genes were detected within the first hour (10–59 minutes) of sequencing. By conventional microbiological methods the presence of ESBL or MRSA would not have been detected until 1–2 days later, a delay which in the setting of BSIs can be fatal. We have also shown that, for most of the samples one to five hours of sequencing was enough to cover 95% of the target species genome. Also, even when more than half of the generated reads were of human origin, eight hours of sequencing was enough.

For mixed cultures the sequencing-based approach performed well when bacteria were present in approximately equal amounts, but for the *E. coli* and *S. aureus* experiment, where there was a 4-log difference in bacterial concentration in favour of *E. coli*, identification of both bacteria and AMR-encoding genes was challenging. After 10 minutes of sequencing 95% of reads were classified as *E. coli* and only 0.05% as *S. aureus*. The *bla*_CTX-M_ gene of *E. coli* was detected in the first sequencing file, but the *mecA* gene of *S. aureus* was only detected after 16 hours of sequencing when ca. 25% of its genome was sequenced. Diagnosing mixed infections, however, is also problematic using conventional microbiological methods. Analysis by MALDI-TOF of pelleted blood-cultures is often unsuccessful in these cases, and overnight sub-cultures on solid culture-media are required for identification from bacterial colonies. Phenotypic AST may take 3 days, since a monoculture is required before AST can be performed.

Raw nanopore reads allow for rapid detection of the *bla*_CTX-M_ and *mecA* genes, but not further subtyping of *bla*_CTX-M_ gene-variants. However, by use of *de novo* assembled nanopore contigs we can correctly identify both plasmids and *bla*_CTX-M_ subtypes, thereby increasing the precision level substantially. This approach, possibly in combination with rapid bacterial sequence typing^22^ harbours great potential for future health care surveillance purposes. For Illumina the time taken from DNA extraction to normalized library was ~5.5 hours when using the Nextera XT DNA library preparation kit as opposed to ca. 3 hours needed for Nanopore library preparation. The additional time to be used for the sequencing by synthesis is very much dependent on the read length and in our case with a maximum read length of 2 × 300 bp, a runtime lasted about 56 hours. Although we kept MinION runs for up to 68 hours, the crucial difference between these two platforms is that in case of Nanopore sequencing, the data become available in real-time, whereas in case of Illumina, one has to wait until the end of the sequencing run. We are aware of very few studies which have applied nanopore whole genome sequencing for blood-culture diagnostics^15,16,23^. Recently, Sakai *et al*.^16^ applied multiple samples on each flow cell by use of barcoding and collected sequencing data for 30 minutes, thereby obtaining 100–3000 reads per sample only. Analysis was done using the ONT analysis software WIMP, which utilizes Centrifuge. The authors report the top three bacteria with the highest read count, and the species with the most assigned reads was used for diagnosis. This approach performed well for Gram negative bacteria but performed poorly for Gram positive bacteria. The study supports the feasibility of rapid nanopore-based identification of pathogens from blood cultures with a minimum of laboratory requirements. Based on our data however, we believe this approach does not generate enough data per isolate for reliable detection of all relevant AMR-encoding genes present in the sample.

It should be possible to develop a bioinformatic analysis pipeline that assembles nanopore data as they become available, performs plasmids search and AMR search against a database of clinically important AMR-encoding genes, and alerts the clinical microbiologist to important findings with very high precision. To implement such an approach in a routine clinical microbiology laboratory the development of a graphic user interface is a necessity, with the most basic information regarding bacterial identity and AMR presented in an easy-to-read fashion^13,14^. More advanced analysis of virulence factors, sequence types, phylogeny and plasmids would require bioinformatic skills. With the rapid development of sequencing-technology, computational power and bioinformatic tools, we believe that such an approach may be the future of routine analysis in clinical microbiology. Furthermore, costs of sequencing are decreasing steadily and flow cells suitable for on-demand sequencing of bacterial genomes have reached the market. Currently, the price per Flongle flow cell, which according to the manufacturer can deliver up to 1–2 gigabytes of data, is $90 (https://nanoporetech.com/). By use of barcoding it should be possible to analyse several samples per flow cell, thereby reducing sequencing-costs to a level that is comparable to other molecular tests which are available in many routine microbiology laboratories.

**Figure 6:**
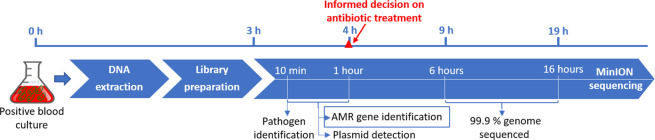
Timeline for the information gathered from nanopore sequencing of positive blood cultures.

In conclusion, we have shown that with a sequencing-based approach to blood culture diagnostics it is possible to identify pathogens and specific AMR-encoding genes using raw nanopore sequencing data, obtained within four hours after a blood culture is flagged as positive by the incubation system. Identification of pathogens was possible after 10 minutes of real-time sequencing, and all predefined AMR-encoding target genes and plasmids from the monoculture experiments were detected within one hour (Fig. 6). Furthermore, we demonstrate correct identification of plasmids and *bla*_CTX-M_ subtypes using *de novo* assembled nanopore contigs. Results from this study hold great promise for future applications in clinical microbiology and for health care surveillance purposes.

## Methods

### Bacterial strains

*E. coli* strain CCUG17620 and *S. aureus* strain CCUG35600 were obtained from Culture Collection University of Gothenburg, Sweden. *E. coli* strain NCTC13441 produces CTX-M-15 ESBL and was purchased from the culture collection at Public Health England and selected due to the availability of whole genome sequence data. *E. coli* strain A2-39 (*bla*_CTX-M-gr.2_), *Klebsiella pneumoniae* strain A2-23 (*bla*_CTX-M-gr.1_), and *K. pneumoniae* strain A2-37 (*bla*_CTX-M-gr.9_) were provided by the Norwegian National Advisory Unit on Detection of Antimicrobial Resistance (K-Res), University Hospital of North Norway, Tromsø.

### Inoculation and incubation of blood cultures

For incubation of blood cultures the BACTEC (BD) system was used. Briefly, bacterial strains were grown overnight on agar plates and suspended into saline at a density of 0.5 McFarland units. The suspensions were diluted with saline to 10^−6^, and 500 µl of the bacterial dilution together with 5 ml untreated human blood were added to one BD BACTEC Plus Aerobic medium flask. Human blood was obtained from healthy anonymous donors via the blood bank at Oslo University Hospital. The flasks were incubated in a BD BACTEC FX blood culture instrument overnight and growth was confirmed by the system. Samples from the cultures were directly used for DNA extraction and dilutions were plated on agar plates for CFU counting after overnight incubation.

### Extraction of bacterial DNA from blood culture and nanopore sequencing

For extraction of bacterial DNA to be used for nanopore sequencing two commercial systems were used, the QIAamp BiOstic Bacteremia DNA Kit from Qiagen (Germany) and the MolYsis Plus kit from Molzym (Germany). DNA extraction was performed according to the manufacturer’s instructions. Purified DNA was then prepared for nanopore sequencing using the Rapid Barcoding Sequencing kit SQK-RBK004 (Oxford Nanopore, UK) following the manufacturer’s protocol. The optional purification and concentration step with the Agencourt AMPure XP system (Beckman Coulter, USA) was included into the procedure. Sequencing was performed on MinION flow cells (R9.4.1 FLO-MIN106, Oxford Nanopore) and data collected using the MinKNOW software v3.6.5 (https://nanoporetech.com/nanopore-sequencing-data-analysis). Basecalling was done online through the EPI2ME service provided by Metrichor (UK).

### Extraction of bacterial DNA and Illumina sequencing

DNA for Illumina sequencing was prepared using the CTAB method described elsewhere^24^. DNA concentrations and purity were determined using the NanoDrop One system and the Qubit 3.0 system with dsDNA HS assay kit (Thermo Fisher Scientific, USA). Library preparation was performed using the KAPA HyperPlus Kit from Kapabiosystems (USA) and adjusted to a final fragment length of about 600 bp. Adapters were NEXTflex DNA barcodes from Bioo Scientific (USA). Sequencing was done for 601 cycles on a MiSeq system using the MiSeq reaction kit version 3 at the Norwegian Sequencing Centre.

### Nanopore sequencing data analysis

The MinKNOW platform generates sequencing data on the fly, outputting 4000 sequences per file using default settings. The first output file is produced approximately ten minutes after the start of the sequencing run. For this work each output file was processed separately keeping track of the time passed from the start of the sequencing. Combined sequencing reads from each complete run were *de novo* assembled using the Unicycler^25^ assembly pipeline setting–min_fasta_length flag to 500 bp. Reads recognized as generated from human DNA were omitted from further analysis and discarded.

### Taxonomy classification

In addition to the online WIMP v3.2.1^18^ ONT analysis tool, raw sequencing reads (≥ 300 bp) were also taxonomically classified by Centrifuge 1.0.4 using default settings (minimum length of partial hits *min_hitlen* = 22; at most *k* = 5 distinct assignments for each read; no preferred/excluded taxa) and bacteria, archaea, virus and human indexing scheme (release 12.062016.)^17^. The output was summarized using the *centrifuge-kreport* command. Additionally, we used the BLAST search algorithm with the NCBI RefSeq (release 93, 16.03.2019) and NCBI Prokaryotic RefSeq (RefProk, release 18.10.2018) databases. Only hits with ≥ 85% similarity, E-value ≤ 10^−6^ and with ≥ 80% coverage were kept. The ANI was calculated using the orthoANIu online calculator^26^.

### Plasmid detection

Raw sequencing reads (≥ 300 bp) were searched against the comprehensive plasmid sequences database from Brooks *et al*.^19^ using BLAST (downloaded March2019), Only hits with ≥ 80% similarity, E-value ≤ 10^−6^ and with ≥ 80% coverage of the query were kept. Assembled contigs were initially searched against the NCBI plasmid database using the PlasmidFinder v.2.0.2^27^ and positive hits were additionally confirmed through the search against the PLSDB plasmid database v.2019_06_03^28^.

### AMR genes search

Raw sequencing reads (≥ 300 bp) and assembled contigs tagged as plasmids, were searched against the nucleotide-based CARD (v3.0.1, release February2019)^29^ and ResFinder (release February 2019)^30^ databases using BLAST. Only hits with ≥ 80% similarity, E-value ≤ 10-6 and with ≥ 50% coverage of the database entry were kept.

### Illumina sequencing data analysis

Raw sequencing reads were read error corrected and assembled using SPAdes v.3.13.1^31^. For taxonomy assignment, corrected unassembled reads were classified by Centrifuge 1.0.4 as described above. Assembled contigs were searched against the RefProk database using BLAST (contigs ≥ 10000 bp; hit length ≥ 10000 bp; similarity ≥ 85%). For plasmid and AMR gene search assembled contigs were analyzed using PlasmidFinder, CARD and ResFinder databases as described above.

### Hybrid assembly, other software

Additionally, we used Unicycler for de novo hybrid assembly of combined Illumina and nanopore reads using the “–min_fasta_length 500” flag and performed plasmid and AMR gene search as described above.

### Genome coverage analysis

Each output file from the nanopore sequencing was BLAST searched against the reference genome sequence using 90% of the similarity threshold. The coverage was calculated as the proportion of the genome that reads were mapping towards. BLAST results were summarized in additive fashion where for each time point *n* all previous output files (*n-1, n-2*,…*1*) were also taken. Hybrid assembly was used as the reference for *E. coli* A2-39, *K. pneumoniae* A2-37 and *K. pneumoniae* A2-23; nanopore assembly – for *S. aureus* CCUG35600 as this strain was not sequenced on Illumina platform. Genome sequence of *E. coli* NCTC13441 was downloaded from the ENA database (WGS project UFZF01) and of *E. coli* CCUG17620 (CP009072) - from the NCBI database.

All results were summarized and visualized in Matlab R2018b (MathWorks Inc., MA, USA) unless stated otherwise. Assembly graphs were visualized in Bandage^32^.

## Supplementary information

Supplementary information.

## Data Availability

The datasets used and analyzed during the current study are available at ENA https://www.ebi.ac.uk/ena/browser/home under accession number https://www.ebi.ac.uk/ena/browser/view/PRJEB60525.
