# Supplementary material for: Rapid identification of pathogens, antibiotic resistance genes and plasmids in blood cultures by nanopore sequencing
Source: Sci Rep. 2020 May 6;10:7622. doi: 10.1038/s41598-020-64616-x (PMC7203151; doi:10.1038/s41598-020-64616-x)
Supplement: Supplementary file 1 — Supplementary information. [file 41598_2020_64616_MOESM1_ESM.pdf]

Rapid identification of pathogens, resistance genes and plasmids for blood stream infection diagnosis using nanopore sequencing

Arne M. Taxt[1,2], Ekaterina Avershina[2], Stephan A. Frye[1], Umaer Naseer[3] & Rafi Ahmad[2,4,*]

[1]*Department of Microbiology, Division of Laboratory Medicine, Oslo University Hospital, PB 4956 Nydalen, 0424 Oslo, Norway*

[2]*Department of Biotechnology, Inland Norway University of Applied Sciences, Holsetgata 22, 2317 Hamar, Norway*

[3]*Department of Zoonotic, Food- and Waterborne Infections, 0213 Oslo, Norwegian Institute of Public Health, Oslo, Norway*

[4]*Institute of Clinical Medicine, Faculty of Health Sciences, UiT - The Arctic University of Norway, Hansine Hansens veg 18, 9019 Tromsø, Norway*

[*]Corresponding author

rafi.ahmad@inn.no

Supplementary Material

Supplementary Figures

Supplementary Figure 1. DNA extraction results for comparison of the two methods used. Concentrations were measured using the Qubit dsDNA HS assay system. Mean with standard error of the mean (SEM) are indicated by horizontal lines and error bars, respectively.

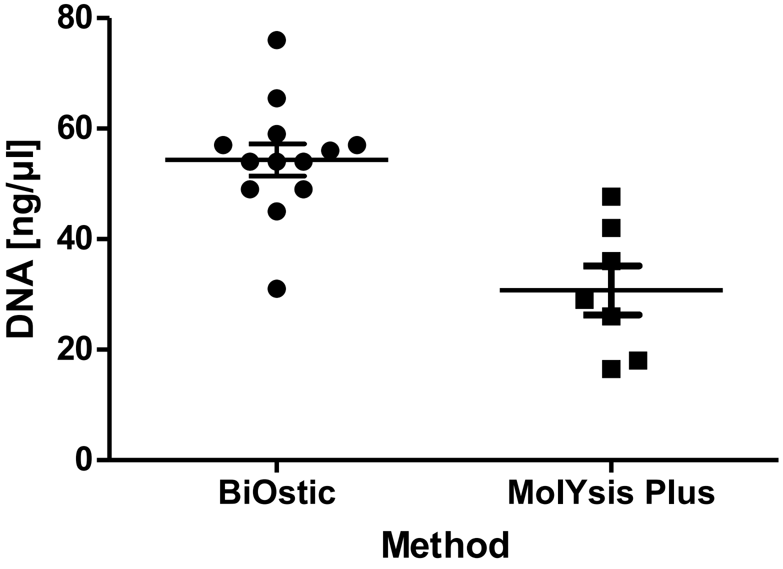

Supplementary Figure 2. Distribution of read lengths of sequence data from nanopore sequencing of DNA purified from blood cultures. Red dashed line denotes typical Illumina single end read length of 300 bp; *n* denotes number of reads with length below/above 300 bp.

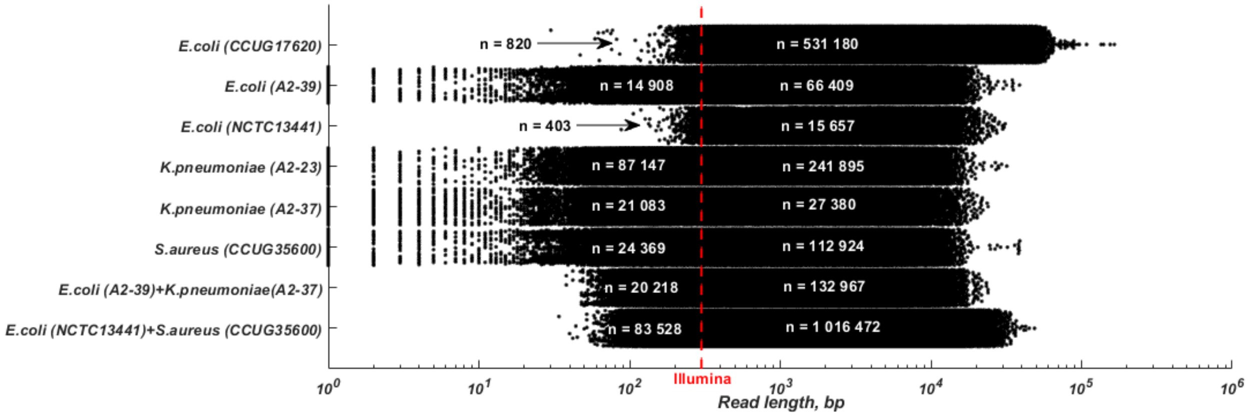

Supplementary Figure 3. BLAST search of the *Salmonella enterica* Centrifuge-assigned reads through the NCBI RefSeq database

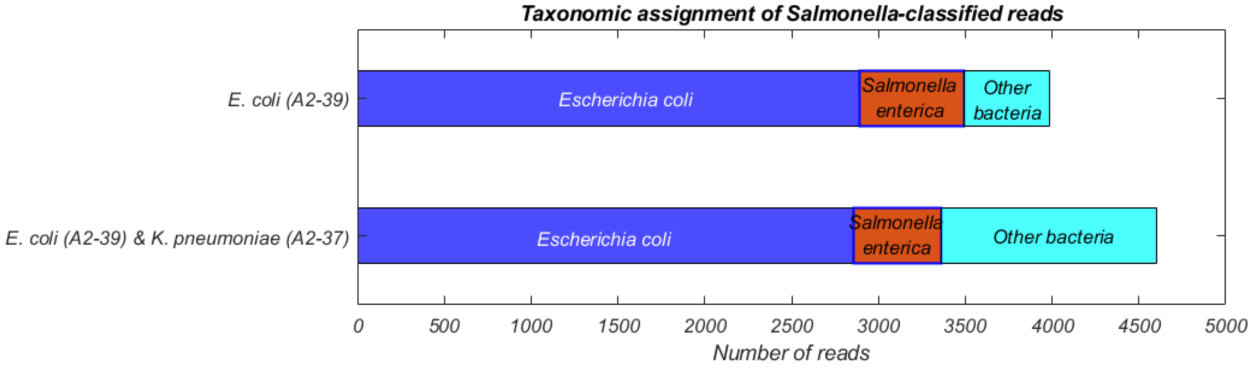

Supplementary Figure 4. Taxonomy assignment of spiked blood cultures. A. BLAST search against the RefProk database of the first output file approx. 10 minutes after the sequencing start. B. Centrifuge assignment of the first 15 output files.

A

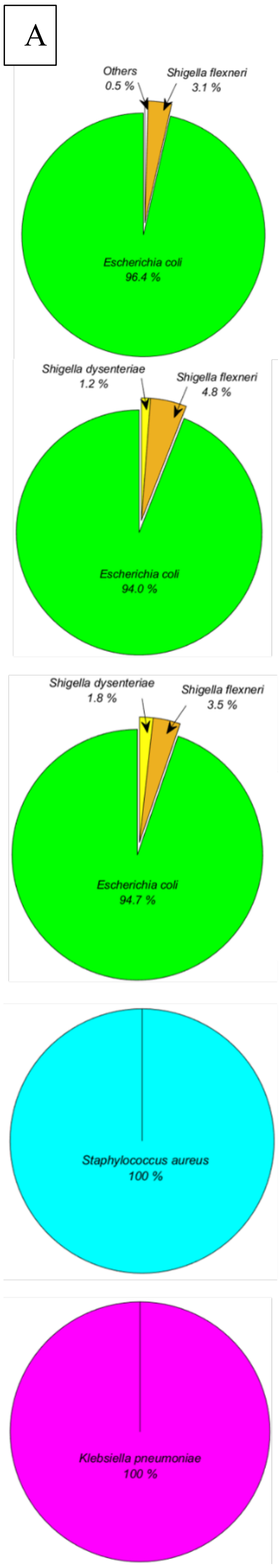

Others
0.5 %
Shigella flexneri
3.1 %
Escherichia coli
96.4 %
Shigella dysenteriae
1.2 %
Shigella flexneri
4.8 %
Escherichia coli
94.0 %
Shigella dysenteriae
1.8 %
Shigella flexneri
3.5 %
Escherichia coli
94.7 %
Staphylococcus aureus
100 %
Klebsiella pneumoniae
100 %

B

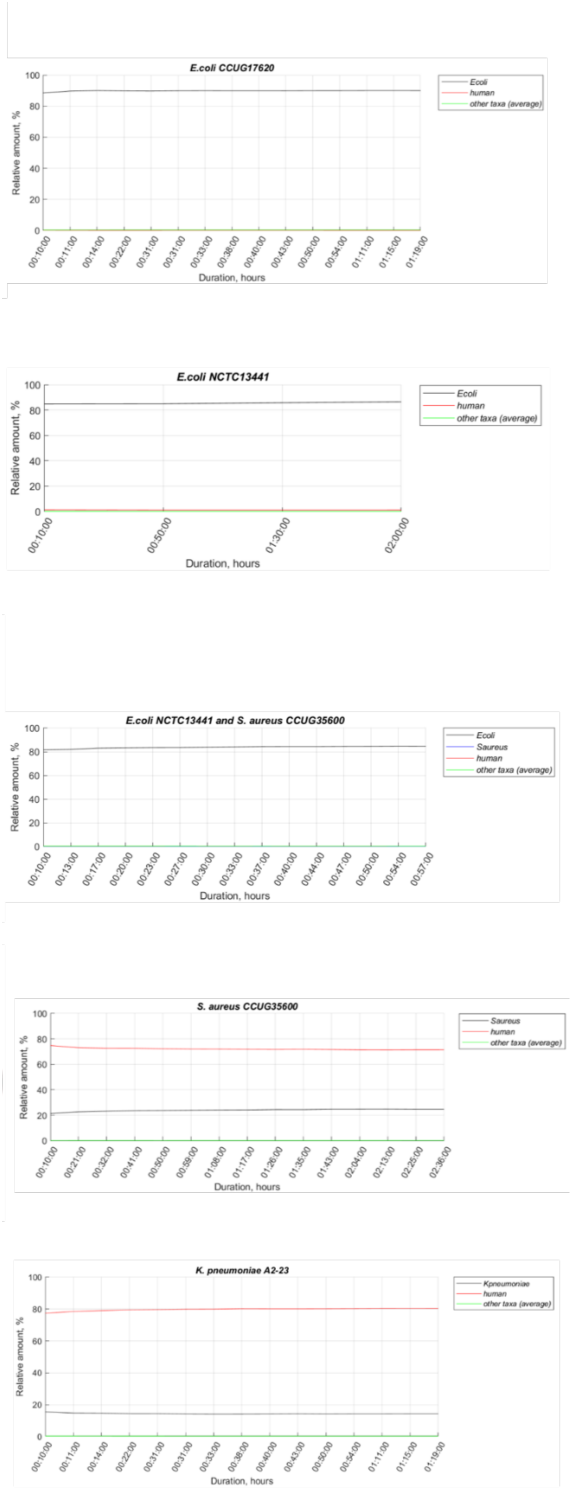

E.coli CCUG17620
Ecoli
human
other taxa (average)
Relative amount, %
Duration, hours
E.coli NCTC13441
Ecoli
human
other taxa (average)
Relative amount, %
Duration, hours
E.coli NCTC13441 and S. aureus CCUG35600
Ecoli
Saureus
human
other taxa (average)
Relative amount, %
Duration, hours
S. aureus CCUG35600
Saureus
human
other taxa (average)
Relative amount, %
Duration, hours
K. pneumoniae A2-23
Kpneumoniae
human
other taxa (average)
Relative amount, %
Duration, hours

Supplementary Figure 5. Number of various CTX-M variants that were detected by BLAST search of the raw MinION sequencing reads through the CARD and ResFinder resistance genes databases. L – length in base pairs (bp); ID – similarity to the database entry, %

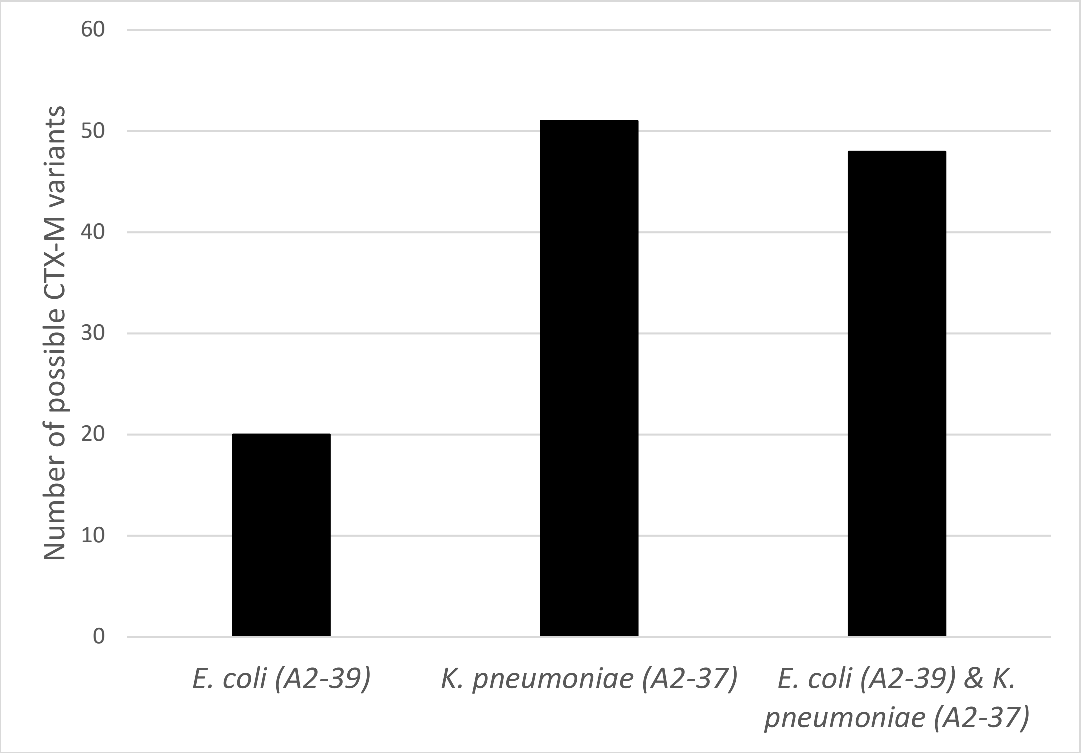

Supplementary Figure 6. Centrifuge taxonomy assignment of Illumina sequencing reads

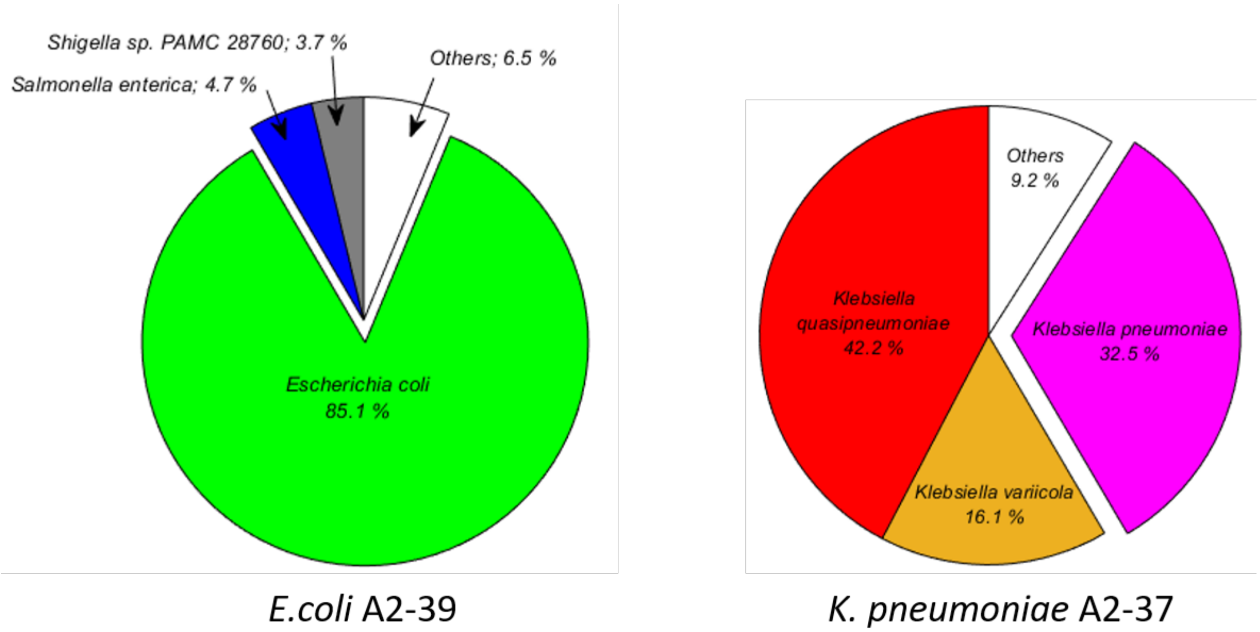

Supplementary Figure 7. Mapping of the *Shigella* identified reads to the hybrid assembly of *Escherichia coli* A2-39

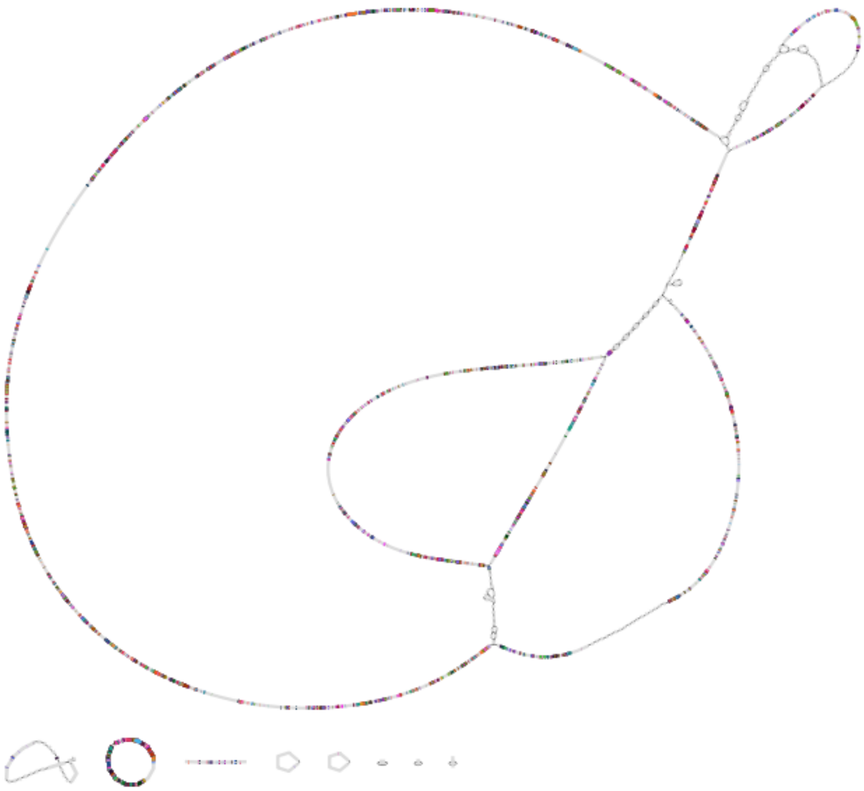

Supplementary Tables

Supplementary Table 1. Assembly statistics

Isolate	Sequencing data	Number of contigs	total length, bp	Longest contig, bp	N50, bp
E. coli **(A2-39)**	MinION	79	6 675 137	2 133 209	714 352
	Illumina	803	6 145 589	203 165	87 318
	MinION+Illumina	60	5 973 956	2 757 734	996 338
K. pneumoniae **(A2-37)**	MinION	42	661 270	59 663	17 750
	Illumina	1763	6 516 794	489 097	173 565
	MinION+Illumina	28	5 562 393	2 952 449	2 952 449
K. pneumoniae **(A2-23)**	MinION	158	4 119 608	123 525	35 892
	Illumina	878	6 208 496	587 610	186 930
	MinION+Illumina	15	5 562 393	2 952 449	2 952 449

Supplementary Table 2. PlasmidFinder search of the *de novo* hybrid assemblies.

Bacterial culture	PlasmidFinder		
	**Plasmid type**	**Query/ HSP length, bp**	**Identity, %**
E. coli **(A2-39)**	*IncHI2*	327/327	100
	IncHI2A	630/630	99.5
	p0111	885/885	98.9
K. pneumoniae **(A2-37)**	*IncFII*	258/261	100
	IncFIA(HI1)	388/388	97.2
	IncFIB(K)	560/560	98.8
